# Possible role of lncRNAs in amelioration of Parkinson’s disease symptoms by transplantation of dopaminergic cells

**DOI:** 10.1038/s41531-024-00661-x

**Published:** 2024-03-12

**Authors:** A. Amini, F. Esmaeili, M. Golpich

**Affiliations:** Department of Plant and Animal Biology, Faculty of Biological Science and Technology, University of Isfahan, Isfahan, Iran

**Keywords:** Molecular biology, Cell biology

## Abstract

Long non-coding RNAs (lncRNAs) are biomarkers for diagnosis and treatment of Parkinson’s disease (PD). Since dopaminergic cell transplantation is a clinical method to treat PD, this study investigated the effects of dopaminergic cell therapy on the expression of some lncRNAs and genes related to PD. In this study, Twenty-eight rats were randomly assigned to four experimental groups. The control group (Sal group) received saline injections. The Par group was a PD rat model with 6-hydroxydopamine (6-OHDA) injection in right striatum (ST). PD animals were transplanted by undifferentiated P19 stem cells (Par-E group), and P19-derived dopaminergic cells (Par-N group). Cell transplant effects were evaluated using behavioral tests (cylinder, open field, and rotarod tests), and histological methods (H&E and Nissl staining, and immunohistochemistry). Moreover, the expression of lncRNAs MALAT1, MEG3, and SNHG1, alongside specific neuronal (synaptophysin) and dopaminergic (tyrosine hydroxylase) markers was evaluated by qRT-PCR. Behavioral and histopathological examinations revealed that cell transplantation partially compensated dopaminergic cell degeneration in ST and substantia nigra (SN) of PD rats. The expression of MALAT1, SNHG1, and MEG3 was decreased in the ST of the Par group, while MEG3 and SNHG1 gene expression was increased in PBMC relative to the Sal group. In PBMC of the Par-N group, all three lncRNAs showed a reduction in their expression. Conversely, MALAT1 and SNHG1 expression was increased in ST tissue, while MEG3 gene expression was decreased compared to the Sal group. In conclusion, dopaminergic cell transplantation could change the lncRNAs expression. Furthermore, it partially improves symptoms in PD rats.

## Introduction

Parkinson’s disease (PD) initially diagnosed by James Parkinson in 1817, is a neurological disorder that affects movement^1^. This disease mostly affects the SN (area A9; the origin of mesostriatal dopaminergic projections to the caudate/putamen complex), where dopaminergic neurons (DA) are destroyed. Dopamine plays an important role in regulating body movements^2^. Therefore, the destruction of this pathway causes disturbances in the basal ganglia circuit which leads to several physical symptoms such as bradykinesia, tremors, and rigidity^1^. Since rodent brain neuroanatomy is almost similar to the human brain, rodent models of PD are remarkable tools to further understand the cause of PD and are also very suitable for scientific research and effective treatment of this disease^3^. Among the neurotoxins used to induce dopaminergic neuron degeneration, 6-hydroxydopamine (6-OHDA), 1-methyl-4-phenyl-1, 2, 3, 6-tetrahydropyridine (MPTP), and rotenone have attracted the most attention. Since 6-OHDA cannot cross the blood-brain barrier, it should be stereotactically and locally injected into the ST, MFB, or SN. 6-OHDA selectively destroys catecholaminergic neurons. It is usually injected unilaterally, which induces asymmetrical rotation behavior in animals^4^.

Today, owing to the pathological characteristics of PD, cell transplantation is used as a promising treatment option for this condition^5^. Embryonic stem cells (ESCs), embryonal carcinoma cells (ECCs), epiblast stem cells (EpiSCs), embryonic germ cells (EGCs), and induced pluripotent stem cells (iPSCs) are all pluripotent stem cells (PSCs)^6^. ECCs are derived from a teratocarcinoma, self-formed in the mouse gonads, or produced by ectopic transplantation of mouse embryos in the early stages^7^. Although ESCs and iPSCs are preferred for investigating the development and functioning of neurons, ECCs have specific advantages. They show less tendency to automatic differentiation and are much more easily maintained in culture^8^. In addition, genetic manipulation of these cells is very easy^9^. Several murine teratocarcinoma cell lines of different tissue origins are available, among which the most well-known are F9 and P19^10^. The P19 cell line was first identified in 1982^11^. P19 cells are capable of differentiating into various cell types, such as nerve cells, glial cells, and microglia^12^. Compared to other cell models, the P19 cell model system has some significant benefits: these cells easily take up and express ectopic genes, showcase a robust proliferation capacity in serum-supplemented media, and can differentiate in large amounts^13^. Due to their advantages, P19 cells have the potential to be a valuable in vitro model for investigating cell differentiation pathways and molecular mechanisms responsible for the pluripotent stem cell differentiation process^14^.

Induction of neural/dopaminergic differentiation was generally applied on the P19 cell line by using natural/synthetic factors including neonatal rat brain extract (NRBE) or RA or a combination of fibroblast growth factor 8 (FGF8) and sonic hedgehog (Shh)^15–18^. Until the late 1970 s, it was widely believed that central nervous system (CNS) treatment would never be possible^19^. Nowadays, cell therapy is proposed as a method of effectiveness in the cure of PD. This method started in 1979 with the transplantation of embryonic mouse DA neurons in Parkinsonian rats, which in addition to the survival of the grafts also caused axonal growth^20^. Results from the mouse and rat models have provided a new treatment approach for patients with PD using the substitution of degenerated and dead dopaminergic cells with normal neurons.

Several intrinsic transcription factors such as Engrailed1/*2 (*En1/2), LIM homeobox transcription factor 1 alpha and beta (Lmx1a/b), forkhead box protein A1and A2 (Foxa1/2(, paired like homeodomain 3 (Pitx3), and nuclear receptor-related factor 1 (Nurr1, also known as NR4A2: nuclear receptor subfamily 4 group A member 2) are involved in the development of dopaminergic cells^21^. Transcription factor Nurr1 lacks a ligand binding site, therefore it acts as a ligand-independent nuclear receptor. Animals that were deficient in Nurr1 lack tyrosine hydroxylase (TH) and other features of DA neurons^22^. Studies have demonstrated that TH gene transcription is regulated by *Nurr1* in neuronal cultures^23^. The reduction in *Nurr1* expression is strongly associated with PD and reduces the survival of DA neurons. The elimination of *Nurr1* in adult mice resulted in axonal abnormalities, degeneration of striatal DA neurons, and Parkinsonian-like behavioral traits^24^. Considering the importance of Nurr1 in PD development, it can be assumed that transplantation of Nurr1-expressing cells to the ST of the patients leads to a.mac009564 reduction in the symptoms of PD.

Among the most important components of the nerve cell membrane are proteins such as Synaptophysin (SYP), Synaptoporin, and *Synaptobrevin*. SYP is a presynaptic marker that is specific for functionally mature neurons and is involved in synapse formation^22^. If these messages are reduced or removed, the nerve cells will die^25–27^. Recently, it has been found that the expression of SYP in nerve cells indicates the presence of functional synapses and the ability to generate action potentials^28,29^. Loss of SYP happens in PD, dementia caused by Lewy bodies, and other neurological disorders. Accumulation of alpha-synuclein (α-Syn) in PD and dementia with Lewy bodies causes loss of SYP in embryonic or post-natal mouse cortical neurons and the neurons of adult mice hippocampus^30^.

Non-coding RNAs (*ncRNAs*), such as long non-coding RNAs (lncRNAs), are involved in processes including gene regulation, cell proliferation and differentiation, transcriptional interference, regulating the expression of imprinted genes, and chromatin modification^31^. lncRNAs are strongly expressed in the CNS^32^. Since the expression of metastasis-associated lung adenocarcinoma transcript1 (MALAT1) is increased in neuropathologically altered brain tissue, it can be assumed that MALAT1 plays a significant role in the pathogenesis of PD^33^. Abnormal MALAT1 expression has been shown in PD^34^. Recent evidence suggested that MALAT1 is involved in neuronal dendritic development and synaptic formation. Furthermore, it was highly expressed in methyl-4-phenyl pyridinium (MPP + )-induced PD cells and brain tissue of 1-methyl-4-phenyl-1, 2, 3, 6-tetrahydropyridine (MPTP)-induced PD mouse model^35,36^. MALAT1 is strongly expressed in brain tissue, especially in very active regions of the human neocortex^36^. Recently, it has been indicated that after the depletion of MALAT1, the specific gene expression patterns markedly related to dendrite development and/or synapse maturation in cultured neural cells have been changed^33^.

Overexpression of the lncRNA maternally expressed gene-3 (MEG3), also referred to as gene trap locus 2 (Gtl2), reduces neuronal activity and inhibits cell proliferation and tumor development^37^. These results indicate that MEG3 can play an important role in CNS epigenetic regulation^38^. Recently, Huang et al. (2021) observed that MEG3 was downregulated in MPP + -induced SH-SY5Y PD cells. On the other hand, overexpression of MEG3 was associated with inhibiting apoptosis in cells treated by neurotoxin MPP + ^39^.

Small molecule RNA host gene 1 (SNHG1), also known as linc00057, is a cancer-associated lncRNA that plays a role in the emergence and development of various malignancies^40^. A new study showed that SNHG1 has a role in regulating brain function and CNS disorders^41^. In addition, it was markedly enhanced in the brain samples of PD patients and SH-SY5Y cells exposed to 6-OHDA and MPP + ^42^.

**Fig. 1:**
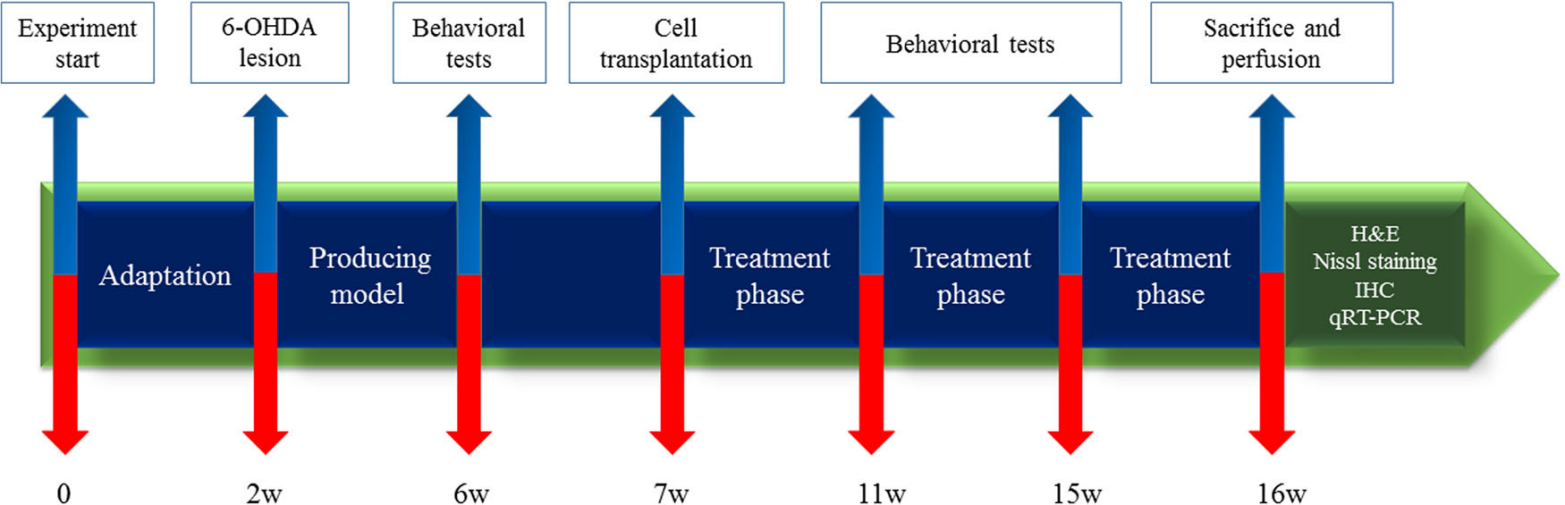
The experimental design of the present study. The rats were divided into four main groups. A line of embryonal carcinoma stem cells, P19, was differentiated into dopaminergic neurons and injected into the animals. Behavioral tests were performed both before and after cell transplantation. The experiment lasted 16 weeks. Finally, animals were euthanized and brain samples were collected for qRT-PCR and histopathological analysis.

Our previous studies showed that Nurr1-transfected P19 cells exposed to neonatal rat brain extract (NRBE) more efficiently differentiated into dopaminergic cells and expressed some neuronal and dopaminergic-specific markers^18^. Therefore, in the present study, we transplanted these differentiated cells into a PD rat model. The animals were assessed for behavioral changes, after cell transplantation. The brain specimens from ST and SN were analyzed histologically using specific histological methods. Furthermore, the expression of some lncRNAs as well as neuronal and dopaminergic markers was evaluated (Fig. 1).

## Results

### Cylinder test

**Fig. 2:**
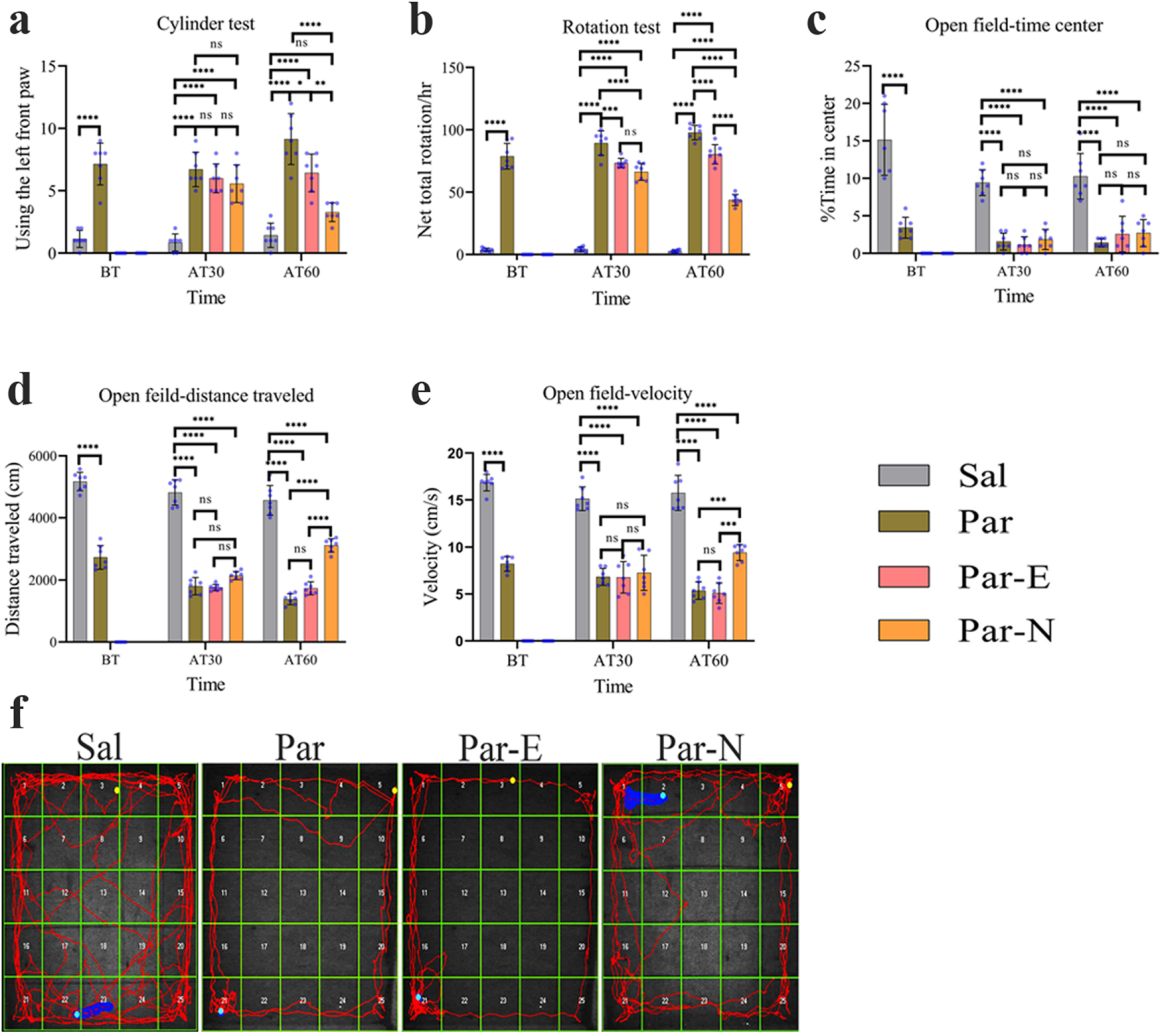
Behavioral tests after unilateral 6-OHDA-induced intrastriatal lesion and cell transplantation. The following behavior tests including cylinder, rotation, and open field tests were taken from the rats at the time of lesion induction, and 30 and 60 days after the surgery. Statistical analysis exhibited a significant difference between the groups 30 and 60 days after transplantation. **a** The cylinder test exhibited a significantly increased number of wall touches of the contralateral front paw (the opposite side of the lesion) on the cylinder wall in Par, Par-E, and Par-N groups (*n* = 7) as compared to the Sal group. On the other hand, the Par-N group relative to Par and Par-E groups exhibited a significant reduction in using the left front paw. **b** Rotation test diagram after apomorphine administration. Par-N exhibited lower contralateral rotations compared to the Par and Par-E group. **c** The time spent in the center of the field showed no differences in Par-E and Par groups. These groups had a significant difference compared to the Sal group 60 days after transplantation. **d** The analysis of the open field-distance traveled test indicated a significant decrease in distance traveled in all the experimental groups compared to the Sal group. **e** Velocity in the Par, Par-E, and Par-N groups was significantly reduced compared to the Sal group. Nevertheless, the Par-N group displayed a significant increase in contrast to the Par and Par-E group (*P* < 0.001*).* **f** Maps indicative of rat activity after 60 days. All the data of these experiments were mentioned as the mean ± standard deviation (SD) (*n* = 7). One-way ANOVA was used to evaluate the differences between the mean counts of the groups. *P* values less than 0.05 (*), 0.01 (**), 0.001 (***), and 0.0001 (****) were regarded as statistically significant. BT before transplantation, AT30 30 days after transplantation, AT60 60 days after transplantation, Sal (control: the rats injected with isotonic saline solution); Par (Parkinson’s rat model: 6-OHDAlesioned animals); Par-E (Parkinson’s rats under cell therapy: A line of embryonal carcinoma stem cells, P19 was transfected with pCMV3-GFPSpark vector and injected into the animals); Par-N (Parkinson’s rats under cell therapy: P19 cells were transfected with pCMV3-NR4A2-GFPSpark vector and injected into the animals).

The injection of 6-OHDA was applied to induce striatal lesions in the right hemisphere of the brain. A month afterward, the lesion was determined by the dominant utilization of the ipsilateral front paw, based on the increased counts of limb contacts on the cylinder walls compared to the left one (Fig. 2a). Administration of normal saline in the right hemisphere (Sal group) caused no changes in the expected ordinary utilization of the front paws. The Par-N group showed less use of the left front paw than the Par group, 60 days after transplantation (*P* < 0.0001). Also, in the Par-N group, the use of the left front paws significantly decreased compared to the Par-E group (*P* < 0.01), however, compared to the Sal group, there was no significant difference.

### Rotation test induced by apomorphine (rotarod test)

Apomorphine-induced rotation test was performed after 6-OHDA lesions and cell transplantation as indicated in Fig. 2b. After four weeks, there was a general tendency to an increment turning against the direction of damage in the unilateral right 6-OHDA lesioned animals (Par group) compared to the Sal group (*P* < 0.0001). Sixty days after cell transplantation, the Par, Par-E, and Par-N groups showed a statistically significant increase in the number of rotations against the direction of damage (rotational asymmetry behavior) compared to the Sal group (*P* < 0.0001). Also, rotational asymmetry behavior in the Par-N group decreased significantly compared to the Par and Par-E groups (*P* < 0.0001).

### Open field test

The open field test was applied to evaluate the level of public motor activity in PD rats. The time spent in the center by the animals, distance traveled, and velocity were quantitatively evaluated for all rats in various groups 30 days after the lesion, and 30 and 60 days after cell transplantation (Fig. 2c–e). The results showed that the time spent in the center was less in the Par, Par-E, and Par-N groups compared to the Sal group (*P* < 0.0001). No significant differences existed between Par, Par-E, and Par-N groups in time spent in the center of the field (the “anxiety” criterion) (Fig. 2c). In addition, measuring the distance traveled in the open field test in different groups showed that Par and Par-E groups showed a significant decrease in distance traveled compared to the other groups (*P* < 0.0001). On the other hand, in the Par-N group, the distance traveled decreased significantly compared to the Sal group (*P* < 0.0001), however, compared to the Par and Par-E groups, it increased significantly (*P* < 0.0001) (Fig. 2d). The analysis of the data indicated that velocity in Par, Par-E, and Par-N groups was prominently reduced compared to the Sal group (*P* < 0.0001), however, the Par-N group indicated a marked increase compared to the Par and Par-E group (*P* < 0.001) (Fig. 2e).

### Hematoxylin and Eosin (H&E) staining

**Fig. 3:**
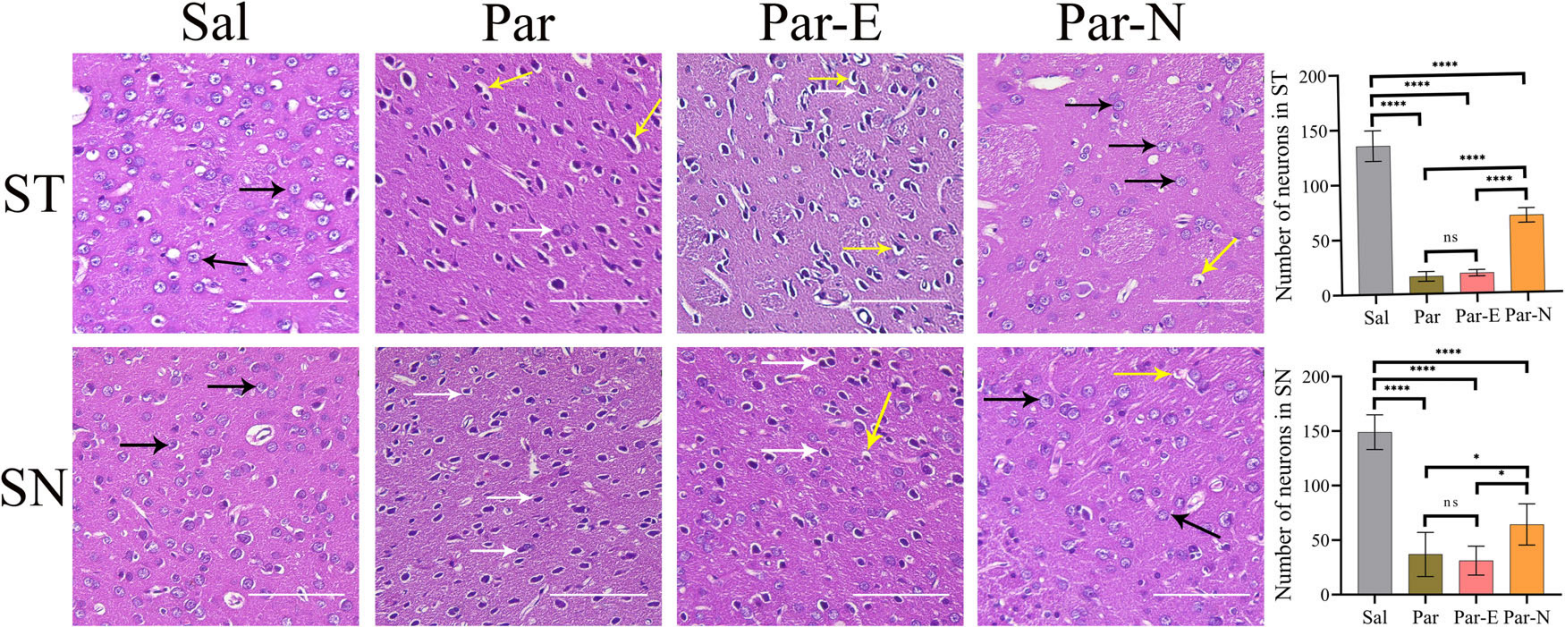
Histological changes in the striatum (ST) and substantia nigra (SN) of the rats, stained with H&E. The tissue sections of the Sal group exhibited normal structure (black arrows). In Par and Par-E groups, destroyed neurons (white arrows), and neuropil vacuolation (yellow arrows) were observed. The destruction of neurons was reduced in the Par-N group. Some neurons indicated degenerative changes and mild neuropilian vacuolization. Furthermore, the number of neurons in the ST and SN in all groups is shown in the graphs (scale bar = 10 μm). All the data were mentioned as the mean ± standard deviation (SD), (*n* = 7). One-way ANOVA was used to evaluate the differences between the mean counts of the groups. *P* values less than 0.05 (*), 0.01 (**), 0.001 (***), and 0.0001 (****) were regarded as statistically significant. Sal control: the rats injected with isotonic saline solution, Par Parkinson’s rat model: 6-OHDA-lesioned animals, Par-E Parkinson’s rats under cell therapy: A line of embryonal carcinoma stem cells, P19 was transfected with pCMV3-GFPSpark vector and injected into the animals, Par-N Parkinson’s rats under cell therapy: P19 cells were transfected with pCMV3-C-NR4A2-GFPSpark vector and injected into the animals.

6-OHDA led to significant cell contraction/morphological changes in the striatum (ST) and substantia nigra (SN) tissues, 30 days after the toxin injection (Fig. 3). The ST tissue of the Sal groups revealed the natural histology of circle-like to elliptic neurons with prominent nucleoli, pale basophilic cytoplasm, and low staining intensity. However, striatal tissues of 6-OHDA-injected rats (Par) and the Par-E group displayed unusual histology with heavily stained erratically shaped neurons with neuropil vacuolation and swollen neurons. The rats in the Par-N group exhibited normal neurons in terms of their number and shape. Furthermore, the number of neurons was markedly decreased in the striatal tissue of the Par and Par-E groups compared to that of the Sal group. On the other hand, the number of neurons in the Par-N group increased significantly compared to the Par group, but compared to the control group it was significantly reduced (Fig. 3). Also, H&E staining exhibited that the neurons in the SN were compacted and regulated tidily in the Sal group, whereas those of the Par and Par-E groups were markedly reduced. In the Par-N group, the cells in the SN zone were somewhat compacted and regulated neatly.

### Nissl substance staining

**Fig. 4:**
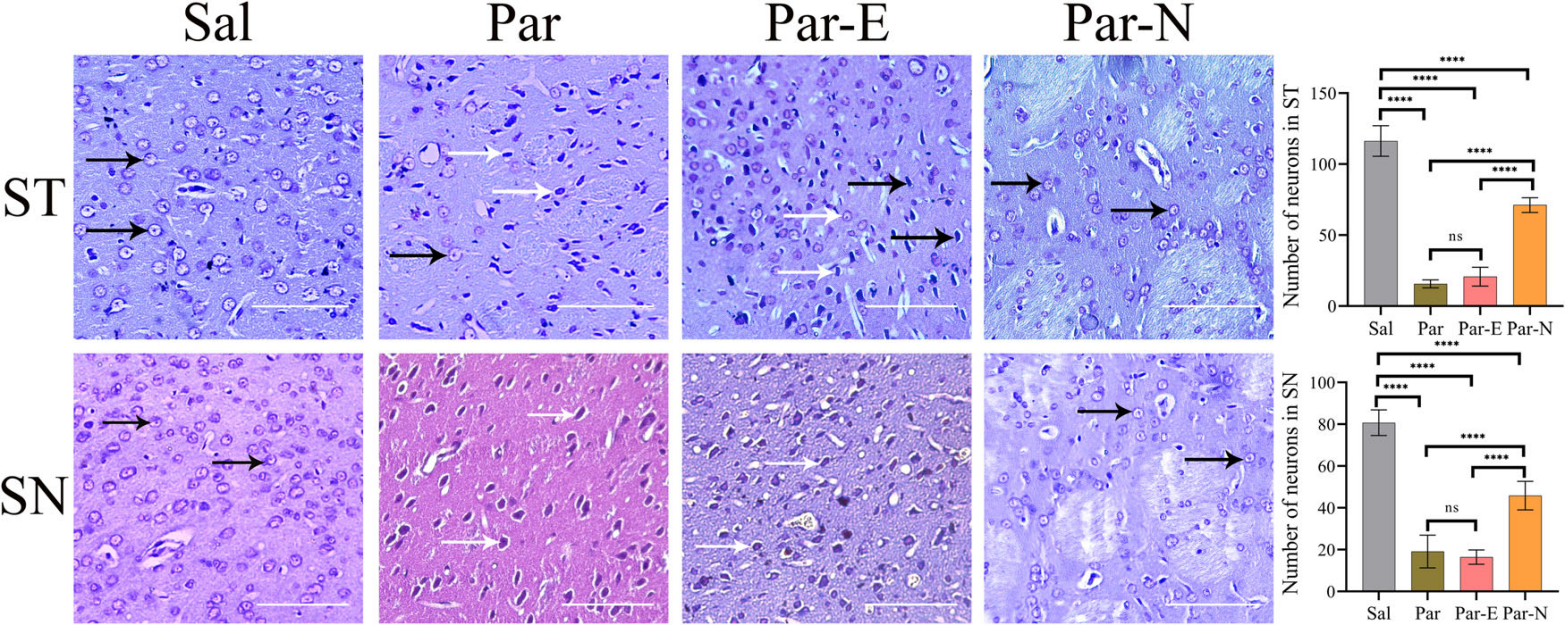
Histological changes in the striatum (ST) and substantia nigra (SN) of rat brain by Nissl *staining*. The ST and SN sections of the Sal group exhibited normal structure. In the Par and Par-E groups, the neurons are severely damaged. In the Par-N group, the Nissl substances were increased and the destruction of neurons was significantly reduced. Normal neurons are shown with black arrows and degenerated neurons with white arrows. Effects of 6-OHDA and cell transplantation on the number of neurons in the SN and ST were also shown in graphs (scale bar = 10 μm). All the data were mentioned as the mean ± standard deviation (SD), (*n* = 7). One-way ANOVA was used to evaluate the differences between the mean counts of the groups. *P*-values less than 0.05 (*), 0.01 (**), 0.001 (***), and 0.0001 (****) were regarded as statistically significant. Sal control: the rats injected with isotonic saline solution, Par (Parkinson’s rat model: 6-OHDA-lesioned animals); Par-E (Parkinson’s rats under cell therapy: A line of embryonal carcinoma stem cells, P19 was transfected with pCMV3-GFPSpark vector and injected into the animals); Par-N (Parkinson’s rats under cell therapy: P19 cells were transfected with pCMV3-C-NR4A2-GFPSpark vector and injected into the animals).

The effect of 6-OHDA and transplanted cells on the histology of the ST and SN of the brain’s right hemisphere in the Sal, Par, Par-E, and Par-N groups were evaluated by Nissl substance staining (Fig. 4). The tissue slices of the Sal group exhibited normal neuronal morphology in the ST and SN. The Par and Par-E groups exhibited strongly stained dark purple neurons with compacted cytoplasm, impaired nuclei, as well as neurons that are almost amoeboid in shape. On the other hand, 6-OHDA-treated rats that received transplanted cells (Par-N) showed markedly decreased Nissl substances and a significant reduction in loss of nerve cells in the ST and SN. Statistical analysis exhibited that the number of neurons with Nissl staining-positive reaction in the striatal zone of the Par group had a significant reduction compared to the Sal group (*P* < 0.001). The number of cells in the Par and Par-E groups was almost similar, however in the Par-N group, the number of Nissl staining-positive neurons compared to the Par group was significantly increased (Fig. 4). Furthermore, Nissl staining exhibited that the number of Nissl-positive neurons in the SN of the animals in the Par group was markedly less than that of the Sal group (*P* < 0.05). There was no significant difference in the neuron number in the SN between the Par-E and Par groups. The number of Nissl staining-positive neurons in the Par-N group was significantly higher than that of the lesion group (*P* < 0.05), while their number in the Par-N group compared to the Sal group was significantly lower (*P* < 0.05).

### Real-time PCR

**Fig. 5:**
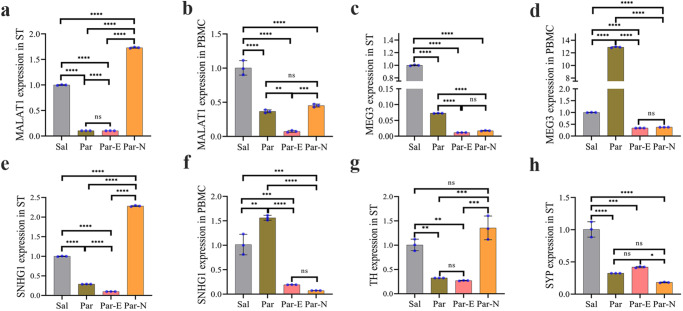
The effect of cell transplantation on the expression of MALAT1, MEG3, SNHG1, TH, and SYP genes in Parkinsonian rats determined by RT-qPCR. **a** MALAT1 expression in ST. **b** MALAT1 expression in PBMC. **c** MEG3 expression in ST. **d** MEG3 expression in PBMC. **e** SNHG1 expression in ST. **f** SNHG1 expression in PBMC. **g** TH expression in ST. **h** SYP expression in ST. All the data were mentioned as the mean ± standard deviation (SD) (*n* = 7). One-way ANOVA was used to evaluate the differences between the mean counts of the groups. *P* values less than 0.05 (*), 0.01 (**), 0.001 (***), and 0.0001 (****) were regarded as statistically significant. Sal (control: the rats injected with isotonic saline solution); Par (Parkinson’s rat model: 6-OHDA-lesioned animals); Par-E (Parkinson’s rats under cell therapy: A line of embryonal carcinoma (EC) stem cells, P19 was transfected with pCMV3-GFPSpark vector and injected into the animals); Par-N (Parkinson’s rats under cell therapy: P19 EC cells were transfected with pCMV3-C-NR4A2-GFPSpark vector and injected into the animals.

The expression level of MALAT1, MEG3, SNHG1, SYP, and TH genes in striatal tissue and peripheral blood mononuclear cell (PBMC) samples of all groups were detected using qRT-PCR. The results showed that MALAT1 expression in the ST of the Par group was lower than that of the Sal group. However, after cell transplantation, the expression of MALAT1 in ST tissues of the Par_N group was markedly higher compared to the Par and Sal groups. In addition, from the comparison of the Par-E and Par-N groups, it was found that the expression of MALAT1 in the Par-E group significantly decreased (*P* < 0.0001) (Fig. 5a). The relative expression level of MALAT1 in PBMCs of the Par group was prominently lower than that of the Sal group (*P* < 0.0001). In addition, there was no significant difference between the Par and Par-N groups in MALAT1 expression. The graph shows that the expression of the Par-E group is significantly lower than that of the Par group (*P* < 0.01). Also, MALAT1 expression in the Par-E and Par-N groups showed a considerable difference compared to the Sal group (*P* < 0.0001) (Fig. 5b).

To assess the expression of the MEG3 lncRNA gene, it was quantified by qRT-PCR. PD induction in the rats led to a reduced expression of MEG3 in the ST at the end of the trial period. The same expression reduction was also observed in the Par-E group. There wasn’t a significant enhancement in MEG3 expression in the Par-N group compared to the Par-E group. However, this group showed a reduction in MEG3 expression compared to the Par group (*P* < 0.0001) (Fig. 5c). The evaluation of PBMC of different blood groups showed interesting results so in the Par group, unlike the striatal tissue, in the PBMC, we had a significant increase in the expression of MEG3 compared to the Sal group (*P* < 0.0001). Whereas, expression of MEG3 was far lower in the Par-E and Par-N groups compared to the Sal group (*P* < 0.0001). On the other hand, cell transplantation in the Par-N group could not increase the level of MEG3 gene expression compared to the Par-E group (Fig. 5d).

To determine if SNHG1 lncRNA was associated with PD and cell transplantation therapy, we measured the expression of this gene in ST and PBMC of the studied groups. In the Par group, in which the dopaminergic cells have been destroyed, the level of expression of this gene in the striatal tissue decreased drastically. Similarly, in the Par-E group, the expression level of this gene was reduced. However, following cell transplantation in the Par-N group, SNHG1 expression was found to increase significantly even more than that of the Sal group (all *P* < 0.0001) (Fig. 5e). SNHG1 gene was also evaluated for expression in the peripheral circulation of the rats. The results showed that its relative circulating level in PBMC of PD rats (Par group) was markedly higher than that of the Sal group (*P* < 0.01). In addition, the expression level of this gene in PBMC of both Par-E and Par-N groups reduced considerably compared to the Par group (*P* < 0.0001). However, its expression did not show any significant difference in the Par-N group compared to the Par-E group (Fig. 5f).

Furthermore, based on the results of qRT-PCR, the expression of dopaminergic-specific gene TH, in ST was significantly diminished in both Par and Par-E groups (*P* < 0.01). On the contrary, after the end of the experimental period, TH expression in the Par-N group was not statistically different from the Sal group (Fig. 5g).

To determine whether transplanted cells were also able to affect synaptic density in PD rats, we investigated the relative expression of SYP in the ST of the animals. In the Par group, the level of SYP expression was found to be lower than that of the Sal group (*P* < 0.0001). The expression of SYP in the Par-N group was not markedly different from the Par group. The expression of this gene in the Par-E group exhibited a significant increase compared to the Par-N groups (*P* < 0.5), While, it failed to increase as much as that of the Sal group (*P* < 0.0001) (Fig. 5h).

### Immunostaining

**Fig. 6:**
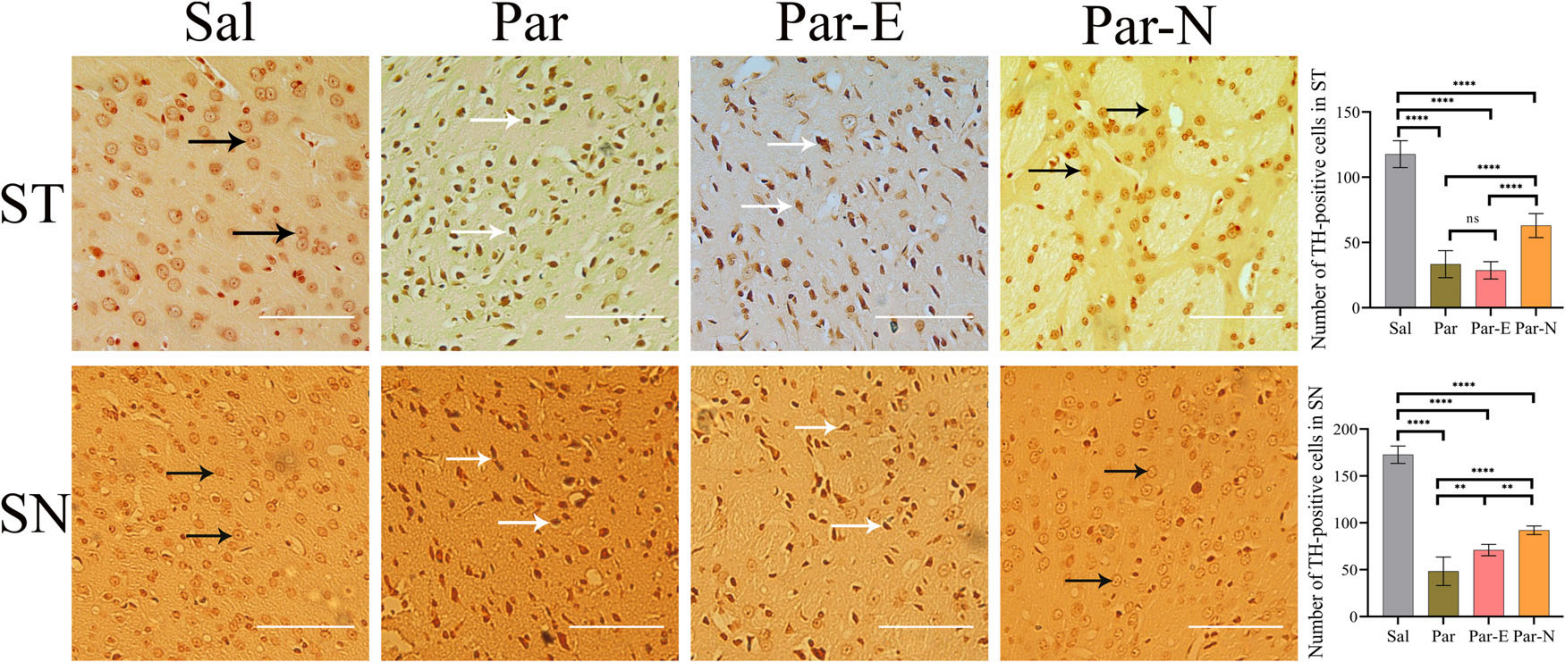
Effect of 6-OHDA injection and cell transplantation on the number of TH+ neurons and cell morphology in the striatum (ST) and substantia nigra (SN) tissues. The Sal group showed normal neurons in both ST and SN (black arrows). The neurons in the Par group were degenerated due to the injection of 6-OHDA in the ST tissue (white arrows). No significant therapeutic effect was observed in the Par-E group. While, in the Par-N group, the tissues were partially normal (white arrows). The graphs indicate the number of TH-positive neurons in the Sal, Par, Par-E, and Par-N groups (scale bar = 10 μm). All the data were mentioned as the mean ± standard deviation (SD) (*n* = 7). One-way ANOVA was used to evaluate the differences between the mean counts of the groups. *P*-values less than 0.05 (*), 0.01 (**), 0.001 (***), and 0.0001 (****) were regarded as statistically significant. Sal control: the rats injected with isotonic saline solution, Par Parkinson’s rat model: 6-OHDA-lesioned animals, Par-E Parkinson’s rats under cell therapy: A line of embryonal carcinoma (EC) stem cells, P19 was transfected with pCMV3-GFPSpark vector and injected into the animals); Par-N (Parkinson’s rats under cell therapy: P19 EC cells were transfected with pCMV3-C-NR4A2-GFPSpark vector and injected into the animals.

The expression of specific markers of dopaminergic neurons TH in the brain tissues was evaluated by immunohistochemistry (IHC) (Fig. 6). The Sal group exhibited normal ST and SN TH+ cells with nuclei of normal healthy neurons. However, the tissues of ST and SN of the Par group displayed significant neuronal degeneration. The neurons appeared as dense and darkly stained cells and their overall number was decreased. The ST and SN of the Par-E group had the same neurodegeneration as the Par group. While in the Par-N group, these tissues showed normal neurons based on their size and structure.

To confirm the effectiveness of the lesion induced by 6-OHDA, the rate of degeneration of dopaminergic neurons in the ST and SN was quantitatively analyzed by TH-immunostained tissues, 60 days after cell transplantation (Fig. 6). The results showed that total striatal TH-immunopositive cell numbers in Par rats were significantly reduced compared to the Sal rats (*P* < 0.0001). On the other hand, the number of TH+ cells in the rats that received transfected cells (Par-N) was significantly higher than that of both the Par and Par-E groups (*P* < 0.0001), although it did not yet reach the number of the control group (Sal). These results indicated that cell transplantation had the capability to improve the pathological damages in the ST zone of the 6-OHDA-induced rat model of PD.

## Discussion

PD is the second most common neurological degenerative disease after Alzheimer’s disease (AD), with a prevalence of about 14 cases per 100,000 people^43^. Studies suggest that unilateral microinjection of the toxin 6-OHDA into the ST and SN can induce an animal model of PD^44,45^. Injection of 6-HODA in rats has been extensively utilized to produce a human PD model. In these animal models numerous pathological characteristics, such as neurodegeneration and a marked reduction of the nigrostriatal DA neurons, alteration of gene expression, and motor malfunction can be reproduced. Such symptoms were confirmed through some behavioral, histological, and molecular assessments. In the present study, histological data from the Par group demonstrated neuro-necrosis in the ST and SN. In addition, immunohistochemical analysis showed a prominent reduction in TH-positive neurons in the ST and SN. Our observations correspond to the earlier studies that reported a marked decline in motilities, gait abnormalities, and a 50% decrease in the activity of TH in the SN^46–48^. It is interesting to note that in this study, H&E staining of the ST and SN exhibited serious histopathological changes including degeneration and reduction of DA neurons in these areas. Furthermore, gliosis was observed in the ST and SN tissues of the rats treated with 6-HODA (data not shown). This phenomenon is a significant neuropathological characteristic of numerous central nervous system disorders^49^. Gliosis and reactive microglia, which indicate an ongoing inflammatory process, are seen in the SN of PD patients^50,51^. Parkinson’s rats treated with 6-OHDA (Par group) also exhibited a marked weight loss relative to the Sal and Par-N groups. This weight loss might be due to a reduction in dietary intake and gastrointestinal (GI) motility disorders. The results of behavioral tests, analysis of tissue sections, and gene expression in the present study, showed that transplantation of P19 cells transfected with pCMV3-C-NR4A2-GFPSpark vector, to the model of Parkinson’s rats, could partially mitigate the disease symptoms. It could ameliorate 6-HODA-induced neurodegeneration in the ST and SN. Preclinical research has shown that dopaminergic neurons retrieved and enhanced motor performance when hASCs (human adipose tissue-derived stem cells) were transplanted into PD models induced by neurotoxins^52,53^. So far, the therapeutic mechanism of P19 cell transplantation is still unclear. However, it is possible that the factors expressed by the differentiated P19 cells markedly improve the neurodegenerative effects of toxins in PD models. It has been previously shown that when exposed to various inducers, P19 cells were able to express different factors including nestin, SYP, TH, Onecut (one cut homeobox 1), and neurotrophic factors (NTF) such as BDNF (brain-derived neurotrophic factor), NGF *(*nerve growth factor), and GDNF (glial cell line-derived neurotrophic factor)^17,18^. Furthermore, these cells can produce and release a highly specific marker of DA cells, TH, in their differentiated form (unpublished data). These all may have positive effects on DA neurons through neuroprotective and neuroregenerative properties. Enhanced expression of NTF protects neurons from oxidative stress, toxicity, and apoptosis^54^. Earlier research has demonstrated that the use of BDNF in animal models of PD prevented DA neuron loss in the ST and SN^55,56^. GDNF has a positive correlation with the survival rate of DA neurons of the nigrostriatal pathway in rodent models of PD^57^. It has been reported that nestin is a neural precursor marker whose expression increased in the ST and astroglial cells of Parkinsonian mice^58–60^. It is possible that activated astroglial cells expressing nestin, in part by synthesizing and releasing BDNF, could play an important role in the protection or degeneration of nigrostriatal DA cells^61^. An experimental study demonstrated that several markers, such as TH and nestin, were expressed on the site of MSCs transplantation^62^. P19 cells are capable of differentiating multi-directionally to generate different kinds of cells for the substitution of missing or dead cells. These cells possess the capacity to differentiate into neural and glial cells, and cells of fibroblast-like in the presence of retinoic acid (RA)^13^. In recent studies, it has been determined that activated astrocytes increase the probability of survival and activity of dopaminergic cells and repair brain damage by using bFGF *(*basic fibroblast growth factor), which is necessary to regulate the differentiation of stem cells into dopaminergic neurons^63^. TH is a specific marker for DA neurons which can, somewhat indicate the number of DA neurons in the brain tissue section. The major significant pathological alteration in PD is the death of DA neurons in the SN and therefore, the reduction of dopamine in the striatal tissue^64^. Chen et al. (2017) exhibited reduced levels of TH expression in the brain tissues of PD rats, which can be linked to the incidence and progress of PD^65^. These findings have increased this hope that PD was partly related to a lack or defect of TH and stimulation of TH may offset functional impairment. In this research, we observed that decreased expression of striatal TH was associated with abnormal morphology of neurons and neuronal degeneration. On the contrary, cell transplantation in Parkinsonian rats could increase the expression of the TH gene. One of the specific presynaptic markers of neurons we studied was synaptophysin, which has been related to cognitive impairment in neurodegenerative diseases^66^. SYP is a key component involved in synaptic vesicle recycling and synapse formation and as a synaptic vesicle transmembrane protein makes structures similar to gap junctions^67^. Yang et al. (2020) reported that the expression level of the SYP gene in PD and *levodopa-induced dyskinesia* (LID *(*rats decreased markedly compared to the control rats^68^. Our studies showed a decrease in SYP levels in PD rats, while its expression increased in the cell transplantation group compared to the Parkinsonian group. Interestingly, it did not show a significant difference in comparison to the saline group.

LncRNAs are important biological markers for the pathogenesis of PD discovery and treatment approaches^31^. Some recent studies have reported that *lncRNAs* such as MEG3, MALAT1, and *SNHG1* are involved in the initiation and progression of PD^39^. There is plenty of MALAT1 in the brain, which is essential for controlling synaptic density^69^. Based on the findings of this research, in the Par group, the expression of MALAT1 decreased in ST and PBMS. However, cell transplantation was able to increase its expression in the ST. These changes may suggest a potential role for this lncRNA in the development and progression of PD. MALAT1 occurs ubiquitously in almost every human tissue with the most widely expression in the brain, pancreas, and lungs. Although MALAT1 has a vital role in the development of organisms, studies have shown that its deficiency in mice has no phenotypic effects^33,36^. MALAT1 deficiency has been shown to reduce synaptic density in vitro, while its upregulation leads to an improvement in this density^70^. In addition, the expression level of MALAT1 seems to be different in various parts of the brain^71^. For instance, Kryger et al. (2012) found a massive rise in MALAT1 transcription just in the brainstem, hippocampus, and cerebellum of alcoholic people, with no notable enhancement in its expression in the frontal or motor cortex^72^. In contrast to the results of the present study, it has been previously revealed that MALAT1 was increased in the mouse model of MPTP-induced PD and MPP+ treatment cells^73^. Theo et al. (2019) proved in their studies that the expression of lncRNAs, such as SNGH1, MALAT1, and lincRNA-p21, was markedly increased in PD people^74^. Also, Lv et al. (2021) found that the expression of MALAT1 increased in SK-N-SH and SK-N-BE cells treated with MPP + , while depletion of MALAT1 causes cell proliferation and inhibition of apoptosis^34^. At present, we cannot explain the discrepancy between these findings and ours. Since there is limited knowledge about the molecular mechanism of MALAT1, further investigation is needed to determine its exact role in PD. In accordance with our study, it has previously been shown that the expression of MALAT1 in the peripheral blood of patients with lung cancer was reduced compared to that of the control^75^. However, cell transplantation did not change the expression level of MALAT1 in PBMC of Parkinsonian rats, as there was no significant difference between the Par and the Par-N groups.

In the current study, the expression of MEG3 was evaluated in the Sal, Par, Par-E, and Par-N groups. From the results, we have realized that striatal destruction by 6-OHDA led to a reduction of MEG3 expression in all the experimental groups compared to that of the control (Sal group). Therefore, it is suggested that changes in MEG3 expression can be a potential target for PD treatment. lncRNA MEG3 is related to a range of cancers, and neurodegenerative diseases including PD, Huntington’s disease (HD), and ischemic stroke^60,76^. This is suggested that MEG3 is one of the several molecules that could assist in the diagnosis and treatment of PD^77^. It has been shown that the expression level of MEG3 is decreased in cancer cells, and up-regulation of MEG3 may inhibit tumor development. Therefore, its primary function was considered to be as a tumor suppressor^78,79^. In addition, there is a great deal of evidence that MEG3 can influence cell development, growth & differentiation, in vivo^80^. MEG3 is highly expressed in numerous normal tissues such as the brain and pituitary gland^81^. Its expression is either reduced or lost in most cancerous tumors and their derived cell lines. Numerous studies have indicated that MEG3 is related to the beginning, evolution, and metastasis of cancer^80^. In a study, it was found that MEG3 was markedly reduced in people with PD compared to healthy people, highlighting the implication of MEG3 in PD^39^. Accordingly, our results confirmed the above findings regarding the effect of PD situation on MEG3 expression in striatal tissue. However, cell transplantation did not affect the normalizing MEG3 expression and caused a further decrease in its expression. In particular, our findings demonstrated that MEG3 was increased in PBMC of the Par group. Sudhalkar et al. (2018) found during their research that the expression of MEG3 in PBMC was higher in psychosis patients compared to healthy individuals, which is consistent with the results of our experiments. Also, they showed that by administering antipsychotic drugs to the patients, the level of MEG3 expression in the PBMC was decreased^82^. This is consistent with the results of the cell transplantation group (Par-N), where MEG3 expression was effectively downregulated.

Earlier research has provided evidence of the potential involvement of lncRNA SNHG1 in the pathogenesis of PD^42^. It was found that SNHG1 in in vitro PD models improves neuronal apoptosis with miR-153-3p blocking, and promotes the progression of Parkinson’s disease by inhibiting PI3K/Akt^83^. Wang et al. (2021) reported that SNHG1 expression was increased in neuroblastoma cells treated with MPP + , and SNHG1 knockdown inhibited cell death and apoptosis induced by MPP+ in these cells^31^. Kraus et al. (2017) demonstrated that the expression of SNHG1 was higher in the brain specimens of people with PD^84^. SNHG1 has previously been indicated to facilitate PD pathogenesis and progression by impacting abnormal α-Syn aggregation and neuro-inflammation through various mechanisms^85^. The results we have achieved concerning the amount of SNHG1 expression in the ST tissue of the brain of Parkinsonian rats conflict with the above-mentioned research, which requires more research and investigation in this field. However, with cell transplantation, the expression level of SNHG1 was increased in comparison to the normal level. Recent genetic research leads us to believe that the inflammation in PD is mediated by myeloid cells, which include peripheral monocytes/macrophages and microglia^86^. Nissen et al. (2019) showed that monocytes in the PBMC of Parkinson’s patients have a higher proliferative capacity than the control group^87^. Our research showed that the expression of SNHG1 was increased in the PBMC of the Parkinsonian group, while the cell transplantation caused its expression to be below the normal level.

Our experiments provided new insights into possible interactions between cell transplantation and lncRNAs expression. Since rats with PD have been under treatment with dopaminergic cells derived from the differentiation of P19 cells, one could infer that variations in gene expression could be owing to the effects of cell transplantation. Furthermore, we showed that cell transplantation could induce a partial repair of damaged tissues in mouse models of PD. These results can provide a new perspective on therapeutic strategies to enhance the effectiveness of PD treatment. Having done this experiment with other stem cells, the question remains whether these results could be true for cells from other sources. On the other, considering that the precise role of lncRNAs in PD has not yet been determined, further research is needed to elucidate these intricate mechanisms.

## Methods

### Experimental scheme

The animal procedures in the present study were performed according to the rules and regulations set by the Bioethics Committee of the University of Isfahan (Code: IR.UI.REC. 1400.083), based on the National Specific Ethical Guidelines for Biomedical Research issued by the Ministry of Health and Medicinal Education (MOHME) of Iran in 2005. Wistar rat strain was obtained from Isfahan University of Medical Sciences (Isfahan, Iran) and maintained under standard situations (20 ± 2 °C, with a regular dark/light cycle and ad libitum access to food and water). First, 28 male rats (220–280 g) were assigned randomly into four groups (Table 1): (1) Sal (control: the rats injected with isotonic saline solution); (2) Par (Parkinson’s rat model: 6-OHDA-lesioned animals); (3) Par-E (Parkinson’s rats under cell therapy: A line of embryonal carcinoma stem cells, P19 was transfected with pCMV3-GFPSpark vector and injected into the animals); (4) Par-N (Parkinson’s rats under cell therapy: P19 ECCs were transfected with pCMV3-C-NR4A2-GFPSpark vector, containing Nurr1 gene, and injected into the animals). The cells in groups Par-E, and Par-N were already treated by NREB. Briefly, neonatal Wistar rats were euthanized at about one week of age; their brains were taken out and homogenized in a solution that inhibits protease activity. After centrifugation at two speeds: 3000 rpm (10 min) and 12,000 rpm (20 min), the whole NRBE samples were combined, and the protein levels were assessed. Finally, the extract was stored at −70 °C until needed^18^.

**Table 1:** Experimental scheme

Groups	Experimental design
Sal (control: *n* = 7)	0.9%NaCl (5 ml/rats)
Par (Parkinson’s rat model: *n* = 7)	25 mg/kg 6-OHDA
Par-E (Parkinson’s rats under cell therapy: *n* = 7)	25 mg/kg 6-OHDA + cells (1 *×* 10^5^/µl P19 cells transfected with pCMV3-GFPSpark vector)
Par-N (Parkinson’s rats under cell therapy: *n* = 7)	25 mg/kg 6-OHDA + cells (1 *×* 10^5^/µl P19 cells transfected with pCMV3-C-NR4A2-GFPSpark vector)

### Unilateral 6-OHDA lesion model and surgical method

6-OHDA (Sigma Chemical Co, St Louis, MO, USA) was used to engender rat models for PD^88^. To induce sectional or perfect evacuation of the dopaminergic neurons, the toxin was injected stereotaxically into the right ST. In summary, desipramine (25 mg/kg) was administered intraperitoneally, a half-hour before 6-OHDA injection, and the rats were anesthetized via IP injection of ketamine (4.5 mg/kg) and xylazine (40/10 mg/kg). The animals then received an injection of 6-OHDA (5.5 μg/μl, 3 μl/injection site), or an equivalent volume of 0.9% saline containing 0.2% ascorbic acid, in the right ST, at a speed of 1 μl/min. The injection location was coordinated based on bregma (mm) and dura according to Paxinos & Franklin’s atlas: 1 AP, 3 ML, and 5 DV^89^.

### Cell transplantation

The surgical steps were performed under completely sterile conditions. The rats were anesthetized with a mixture of Ketamine (100 mg/kg) and xylazine (10 mg/kg) and put in a stereotactic device. The cell suspension (5 × 10^5^ in 5 μL of normal saline) was administered into the right ST by a 10 μL Hamilton syringe at a fixed speed for 5 min (0.5 μL/min). After the administration, the syringe was permitted to stay at the site for three min and then leisurely pull out for one min. The animals were injected with cyclosporine (50 mg/kg) one day before cell injection, and then daily for 60 days.

### Cylinder test

Since unilateral injection of 6 OHDA can lead to limb disorders, the cylinder test was carried out to check involuntary front limb lateralization. This test benefits the inherent discovery nature of rodents in a new milieu. Briefly, the experiment was carried out as follows: the rats were placed individually in a glass cylinder (diameter 22 cm; height 26 cm), while two mirrors were embedded behind the cylinder. The numbers of impaired and non-impaired forelimb contacts are calculated as a percentage of total contacts^90^.

### Apomorphine induced rotation behavior

The rotarod test is a helpful manner of evaluating hypokinesia in a rat model of PD. This experiment was performed four weeks after the unilateral injection of 6-OHDA into the ST of the animals. The rats were placed in cylinders (44 cm width and 40 cm high) and allowed to acclimate to the environment for 60 min before apomorphine hydrochloride (0.1–4 mg/kg SC) administration. The number of rotations (contralateral and ipsilateral) was counted according to the lesioned side caused by the injection of 6-OHDA^91^.

### Open field test

Automatically locomotor behavior in the rats was evaluated by using the open field test. The animals were acclimatized to the surrounding environment two hours before the test. Then, they were located individually in a quadrangular enclosure (90 × 90 × 25 cm) and retained for 5 min under usual lighting. Movements and trajectories of rats were filmed and analyzed using video tracking software (Columbus Instruments, USA). The box was wiped with water and 70% alcohol after every trial to eliminate the body smell, which could be a sign of movement for the rats in the next assessment^92^.

### H&E staining

Sixty days following cellular transplantation, the rats were deeply euthanized and perfused via 50 ml of saline solution, and then with 2% paraformaldehyde. After collecting the brains and fixation in 10% formalin, the tissues were dehydrated with increasing concentrations of alcohol (70, 80, 90, 95, and 100%; 5 min each). Then they were cleared twice in xylene (5 min each, and eventually were embedded into paraffin. After cutting tissue blocks with a thickness of 5 µm, they were placed in an oven at 75 °C for 45 min. In the next step, the tissues were deparaffinized by two changes of xylene (5 min each) and then rehydrated with decreasing concentrations of alcohol (100, 95, 90, 80, and 70%; 5 min each), and washed in tap water for about 5 min. The tissue sections were stained by hematoxylin solution at about 4 min and laundered with distilled water, differentiated by 1% hydrochloric acid ethanol for about 10 s, and then stained by eosin dye. After dehydration, the sections were exposed to xylene for clearing, and finally mounted, examined, and photographed with a light microscope^93^. To quantify the cells with neuronal phenotypes, five microscopic fields were randomly selected. Minimally five repetitions of cell counts were performed for each group, and the results were displayed in graphs^17^.

### Nissl substance staining

After preparing the tissue sections as explained above, they were immersed in cresyl violet solution (0.25% cresyl violet + 0.8% glacial acetic acid + 0.6 mM sodium acetate)^18^. Thereafter, the sections were rinsed, dehydrated, cleared, mounted for observation, and photographed by an optical microscope^94^.

### IHC technique

After the preparation of tissue sections as described above, indirect immunohistochemical staining for TH was performed. Concisely, ahead of action for the IHC staining procedure, the slides need to be deparaffinized and rehydrated. They were put in xylene (2 changes for 20 min each), and then decreasing the concentration of ethanol (absolute, 90%, 70%, 60%, 5 min each). For antigen retrieval, the slides were exposed to trypsin and washed with PBS, and then permeabilized by 10% normal goat serum (NGS, Sigma, G9023) + 0.3% Triton X-100 to block full non-specific antigen binding sites. The tissues were washed and incubated with the primary anti-TH antibody prepared in rabbit (Abcam, ab6211). After tissue washing in PBS, the endogenous peroxidase enzyme was blocked by 3% hydrogen peroxide (H_2_O_2_) for 10 min. The sections were then washed extensively with PBS and incubated with horseradish peroxidase (HRP)-conjugated secondary antibody (Anti-rabbit IgG-HRP, Sigma A6154) at 37 °C for 2 h. Then the slides were incubated with diaminobenzidine tetrahydrochloride (DAB, Sigma, D8001) to demonstrate the antigen. After incubation and washing the tissue with PBS, they were dehydrated with an increasing series of alcohol (60, 70, 80, 90, and 100%), cleared in xylene, and mounted with coverslips. Finally, the slides were examined and photographed by an optical microscope^95^.

### Real-Time RT-PCR

The rats were deeply euthanized and their ST was separated and frozen by direct immersion in liquid nitrogen swiftly. Furthermore, PBMC was isolated from blood specimens by using Ficoll solution (lymphodex inno-train HL60319). Total RNAs were isolated from tissue and blood specimens by RNX Plus solution for total RNA isolation (Sinnaclon, EX6101). Complementary DNA was synthesized (Parstous, EasyTM cDNA Synthesis Kit, A101161), and real-time PCR was performed (StepOnePlusTM Real-Time PCR System) using SYBR Premix Ex Taq (Ampliqon SYBR green Master Mix High ROX, A325402). The sequence of the primers utilized in the present research is given in Table 2. The relative quantity of each target gene expression was normalized with the housekeeping gene (β-actin) and computed by the comparative Ct method quantification (2^-∆∆Ct^ method)^96,97^.

**Table 2:** The Sequences of Real-time PCR Primer

Gene	Accession number	Primers	Product Size
Synaptophysin (SYP)	NM_009305	F: 5’TGGCCACAGCAGTGTTCGCT3‘	217
		R: 5’ACCCAGAGCACCAGGTTCAGGA3‘	
Tyrosine hydroxylase (TH)	NM_009377	F: 5’TGCAGCCCTACCAAGATCAAAC3‘	103
		R: 5’CGCTGGATACGAGAGGCATAGTT3‘	
*MEG3*	W210911-005	F: 5’TCCAATTTCCCCTCCAACCCA3‘	137
		R: 5‘ TCGTGGAACCTGAGCACAAC3‘	
MALAT1	W210911-005	F: 5’AAAGCCTACATGATTAATGCC3‘	99
		R: 5‘ TTTCAGCACATAATGATCCCT3	
*SNHG1*	W210911-005	F: 5’ATCTCAGGCATTCAAAGGTTC3‘	114
		R: 5’AACTTTCCTGGTACGGCTC3‘	
β-actin	NM_007393	F: 5’CCAACCGTGAAAAGATGACC3‘	124
		R: 5’GAGTCCATCACAATGCCAGT3‘	

### Reporting summary

Further information on research design is available in the Nature Research Reporting Summary linked to this article.

### Supplementary information

REPORTING SUMMARY

## Data Availability

The datasets used in the current research are available from the corresponding author upon request.
