# Supplementary material for: Possible role of lncRNAs in amelioration of Parkinson’s disease symptoms by transplantation of dopaminergic cells
Source: NPJ Parkinsons Dis. 2024 Mar 12;10:56. doi: 10.1038/s41531-024-00661-x (PMC10933336; doi:10.1038/s41531-024-00661-x)
Supplement: Supplementary file 1 — REPORTING SUMMARY [file 41531_2024_661_MOESM1_ESM.pdf]

nature portfolio

Corresponding author(s): Fariba Esmaeili

Last updated by author(s): 2024-1-17

Reporting Summary

Nature Portfolio wishes to improve the reproducibility of the work that we publish. This form provides structure for consistency and transparency in reporting. For further information on Nature Portfolio policies, see our Editorial Policies and the Editorial Policy Checklist.

Statistics

For all statistical analyses, confirm that the following items are present in the figure legend, table legend, main text, or Methods section.

n/a	Confirmed	
☐	☒	The exact sample size (*n*) for each experimental group/condition, given as a discrete number and unit of measurement
☐	☒	A statement on whether measurements were taken from distinct samples or whether the same sample was measured repeatedly
☐	☒	The statistical test(s) used AND whether they are one- or two-sided<br>*Only common tests should be described solely by name; describe more complex techniques in the Methods section.*
☒	☐	A description of all covariates tested
☐	☒	A description of any assumptions or corrections, such as tests of normality and adjustment for multiple comparisons
☐	☒	A full description of the statistical parameters including central tendency (e.g. means) or other basic estimates (e.g. regression coefficient) AND variation (e.g. standard deviation) or associated estimates of uncertainty (e.g. confidence intervals)
☐	☒	For null hypothesis testing, the test statistic (e.g. F, t, r) with confidence intervals, effect sizes, degrees of freedom and P value noted<br>*Give P values as exact values whenever suitable.*
☒	☐	For Bayesian analysis, information on the choice of priors and Markov chain Monte Carlo settings
☒	☐	For hierarchical and complex designs, identification of the appropriate level for tests and full reporting of outcomes
☒	☐	Estimates of effect sizes (e.g. Cohen's *d*, Pearson's *r*), indicating how they were calculated

Our web collection on statistics for biologists contains articles on many of the points above.

Software and code

Policy information about availability of computer code

Data collection	Prism-excel-
Data analysis	ANOVA

For manuscripts utilizing custom algorithms or software that are central to the research but not yet described in published literature, software must be made available to editors and reviewers. We strongly encourage code deposition in a community repository (e.g. GitHub). See the Nature Portfolio guidelines for submitting code & software for further information.

Data

Policy information about availability of data

All manuscripts must include a data availability statement. This statement should provide the following information, where applicable:

- Accession codes, unique identifiers, or web links for publicly available datasets
- A description of any restrictions on data availability
- For clinical datasets or third party data, please ensure that the statement adheres to our policy

The datasets used in the current research are available from the corresponding author upon request, but the author reserves the right to reject the request if it is deemed unreasonable.

Research involving human participants, their data, or biological material

Policy information about studies with human participants or human data. See also policy information about sex, gender (identity/presentation), and sexual orientation and race, ethnicity and racism.

Reporting on sex and gender	The animal procedures in the present study were performed according to the rules and regulations set by the Bioethics Committee of the University of Isfahan (Code: IR.UI.REC. 1400.083), based on the National Specific Ethical Guidelines for Biomedical Research issued by the Ministry of Health and Medicinal Education (MOHME) of Iran in 2005. Wistar rat strain was obtained from Isfahan University of Medical Sciences (Isfahan, Iran) and maintained under standard situations (20 ± 2◦C, with a regular dark/light cycle and ad libitum access to food and water). First, 28 male rats (220-280 g) were assigned randomly into four groups
Reporting on race, ethnicity, or other socially relevant groupings	All authors of the manuscript have adhered to ethical considerations and all of them have read the manuscript and agreed to submit it
Population characteristics	28 male adult Wistar rats
Recruitment	All relevant ethical documentation for the treatments conducted on rats is available.
Ethics oversight	I have complied with all ethical considerations for this research.

Note that full information on the approval of the study protocol must also be provided in the manuscript.

Field-specific reporting

Please select the one below that is the best fit for your research. If you are not sure, read the appropriate sections before making your selection.

☒ Life sciences ☐ Behavioural & social sciences ☐ Ecological, evolutionary & environmental sciences

For a reference copy of the document with all sections, see nature.com/documents/nr-reporting-summary-flat.pdf

Life sciences study design

All studies must disclose on these points even when the disclosure is negative.

Sample size	We planned to expand the research further, but the lack of certain laboratory facilities prevented further progress.
Data exclusions	We hereby declare that all data presented in this manuscript are accurate and complete.
Replication	All results reported in this manuscript are based on the analysis of all past and present research data.
Randomization	All data relevant to tables and figures are clearly and concisely presented.
Blinding	We hereby declare that the authors of this manuscript have fully read and understood the author guidelines and that they are aware that the peer review for this type of manuscript is double-blind.

Reporting for specific materials, systems and methods

We require information from authors about some types of materials, experimental systems and methods used in many studies. Here, indicate whether each material, system or method listed is relevant to your study. If you are not sure if a list item applies to your research, read the appropriate section before selecting a response.

Materials & experimental systems

n/a	Involved in the study
☐	☒ Antibodies
☐	☒ Eukaryotic cell lines
☒	☐ Palaeontology and archaeology
☐	☒ Animals and other organisms
☒	☐ Clinical data
☐	☒ Dual use research of concern
☒	☐ Plants

Methods

n/a	Involved in the study
☒	☐ ChIP-seq
☒	☐ Flow cytometry
☒	☐ MRI-based neuroimaging

Antibodies

Antibodies used

1. anti-tyrosine hydroxylase antibody (Abcam, ab6211)
2. horseradish peroxidase (HRP)-conjugated secondary antibody (Anti-rabbit IgG-HRP, Sigma A6154)

Validation

Product name
Anti-Tyrosine Hydroxylase antibody
See all Tyrosine Hydroxylase primary antibodies
Description
Rabbit polyclonal to Tyrosine Hydroxylase
Host species
Rabbit
Specificity
Immunohistochemical distribution throughout the brain shows that staining is restricted to these neurons that are known to contain the enzyme.
Tested applications
Suitable for: IHC-FoFr, WB, ICC/IF, IHC-Frmore details
Species reactivity
Reacts with: Mouse, Rat
Predicted to work with: Chicken, Chimpanzee, Macaque monkey
Immunogen
Synthetic peptide corresponding to Rat Tyrosine Hydroxylase aa 1-100 conjugated to keyhole limpet haemocyanin (Glutaraldehyde).
Database link: P04177
This antibody has proven useful in staining catecholaminergic neurons. It is a high quality reagent that stains these neurons intensely, including dendritic processes and fine nerve terminals.

The Life Science industry has been in the grips of a reproducibility crisis for a number of years. Abcam is leading the way in addressing this with our range of recombinant monoclonal antibodies and knockout edited cell lines for gold-standard validation.

Eukaryotic cell lines

Policy information about cell lines and Sex and Gender in Research

Cell line source(s): P19 embryonal carcinoma (EC) cell line

Authentication: non of the cell lines used were authenticated.

Mycoplasma contamination: The cell line was not tested for mycoplasma contamination.

Commonly misidentified lines (See ICLAC register): *Name any commonly misidentified cell lines used in the study and provide a rationale for their use.*

Animals and other research organisms

Policy information about studies involving animals; ARRIVE guidelines recommended for reporting animal research, and Sex and Gender in Research

Laboratory animals: The tests were performed on laboratory samples in the Biotechnology Laboratory of Isfahan University.

Wild animals: No genetic alterations were made to the laboratory animals, and all animals were healthy. The controls are mentioned in the results and discussion sections.

Reporting on sex:
1-The experiments conducted demonstrate transparency.
2-The authors of the manuscript will use all results from the manuscript to advance their research goals in the future.

3-An original research manuscript includes all the sections mentioned in a research paper.(Abstract - introduction - method, material - results - discussion - reference)
4-All data in the manuscript were accurately presented using statistical analysis software.
5-This manuscript has not been submitted to any other journal other than the one mentioned.

Field-collected samples	This original research proposal is likely to be useful for authors who plan to conduct research on stem cells and lncRNAs involved in Parkinson's disease.
Ethics oversight	All ethical considerations were conducted by the ethical protocols of the Ministry of Health and Medical Education((MOHME), as published in 2005.

Note that full information on the approval of the study protocol must also be provided in the manuscript.

Plants

Seed stocks	It is not significant
Novel plant genotypes	It is not significant
Authentication	It is not significant
